# Collaborative Interventions for Circulation and Depression (COINCIDE): study protocol for a cluster randomized controlled trial of collaborative care for depression in people with diabetes and/or coronary heart disease

**DOI:** 10.1186/1745-6215-13-139

**Published:** 2012-08-20

**Authors:** Peter A Coventry, Karina Lovell, Chris Dickens, Peter Bower, Carolyn Chew-Graham, Andrea Cherrington, Charlotte Garrett, Chris J Gibbons, Clare Baguley, Kate Roughley, Isabel Adeyemi, Chris Keyworth, Waquas Waheed, Mark Hann, Linda Davies, Farheen Jeeva, Chris Roberts, Sarah Knowles, Linda Gask

**Affiliations:** 1 Greater Manchester Collaboration for Leadership in Applied Health Research and Care, Institute of Population Health and Manchester Academic Health Science Centre, University of Manchester, Manchester, UK; 2 School of Nursing, Midwifery & Social Work and Manchester Academic Health Science Centre, University of Manchester, Manchester, UK; 3 Peninsula College of Medicine and Dentistry, University of Exeter and Peninsula Collaboration for Leadership in Applied Health Research and Care (PenCLAHRC), Exeter, Devon, UK; 4 IAPT North West Programme Field Lead, NHS North West, UK; 5 Lancashire Care NHS Foundation Trust, Preston, UK

**Keywords:** Depression, Diabetes, Coronary heart disease, Primary care, Collaborative care

## Abstract

**Background:**

Depression is up to two to three times as common in people with long-term conditions. It negatively affects medical management of disease and self-care behaviors, and leads to poorer quality of life and high costs in primary care. Screening and treatment of depression is increasingly prioritized, but despite initiatives to improve access and quality of care, depression remains under-detected and under-treated, especially in people with long-term conditions. Collaborative care is known to positively affect the process and outcome of care for people with depression and long-term conditions, but its effectiveness outside the USA is still relatively unknown. Furthermore, collaborative care has yet to be tested in settings that resemble more naturalistic settings that include patient choice and the usual care providers. The aim of this study was to test the effectiveness of a collaborative-care intervention, for people with depression and diabetes/coronary heart disease in National Health Service (NHS) primary care, in which low-intensity psychological treatment services are delivered by the usual care provider - Increasing Access to Psychological Therapies (IAPT) services. The study also aimed to evaluate the cost-effectiveness of the intervention over 6 months, and to assess qualitatively the extent to which collaborative care was implemented in the intervention general practices.

**Methods:**

This is a cluster randomized controlled trial of 30 general practices allocated to either collaborative care or usual care. Fifteen patients per practice will be recruited after a screening exercise to detect patients with recognized depression (≥10 on the nine-symptom Patient Health Questionnaire; PHQ-9). Patients in the collaborative-care arm with recognized depression will be offered a choice of evidence-based low-intensity psychological treatments based on cognitive and behavioral approaches. Patients will be case managed by psychological well-being practitioners employed by IAPT in partnership with a practice nurse and/or general practitioner. The primary outcome will be change in depressive symptoms at 6 months on the 90-item Symptoms Checklist (SCL-90). Secondary outcomes include change in health status, self-care behaviors, and self-efficacy. A qualitative process evaluation will be undertaken with patients and health practitioners to gauge the extent to which the collaborative-care model is implemented, and to explore sustainability beyond the clinical trial.

**Discussion:**

COINCIDE will assess whether collaborative care can improve patient-centered outcomes, and evaluate access to and quality of care of co-morbid depression of varying intensity in people with diabetes/coronary heart disease. Additionally, by working with usual care providers such as IAPT, and by identifying and evaluating interventions that are effective and appropriate for routine use in the NHS, the COINCIDE trial offers opportunities to address translational gaps between research and implementation.

**Trial Registration Number:**

ISRCTN80309252

**Trial Status:**

Open

## Background

Depression is a major global public health challenge. Lifetime prevalence is between 2 and 15% [1], and by 2030 depressive disorders are predicted to be the second leading cause of disease burden and disability worldwide [2]. People with chronic physical illness or long-term conditions (LTCs) are two to three times more likely to have depression than healthy controls [3]. When present with other chronic diseases, depression is associated with significantly greater reductions in health status compared with depression alone, or with single or multiple chronic diseases alone [3].

The presence of physical disease also complicates detection and diagnosis of depression, and can lead to poorer treatment response in people with major depressive disorder [4]. The presence of depression might also account for poorer compliance with medical management of physical disease [5], leading to poorer health status and higher costs in primary care [6]. The healthcare costs attributed to untreated depression are high, and the economic burden of depression is increased in the presence of co-morbidity [7].

Screening and treatment of depression is increasingly prioritized. In the UK National Health Service (NHS), the Quality and Outcomes Framework (QOF) of the general medical services contract has, since 2006, provided incentives to general practitioners (GPs) to screen for depression in people with diabetes and coronary heart disease (CHD) [8]. Additionally, the National Institute for Health and Clinical Excellence (NICE) has published guidelines that incorporate stepped-care models to facilitate the delivery of accessible and effective forms of psychotherapy and anti-depressant treatment of depression in adults [9], including adults with LTCs [10]. Furthermore, the NHS is significantly resourced to improve access to routine, evidence-based, first-line treatment of common mental-health problems for adults of working age by rolling out the Increasing Access to Psychological Therapies (IAPT) program across England.

However, depression remains under-detected and under-treated [11]. Detection rates of depression by non-psychiatric doctors are generally low [12], and only a minority of patients with LTCs attending primary care present with a psychological problem [13]. In the UK, even where case-finding for depression attracts incentives through the QOF, rates of treatment for depression are lower in patients with medical co-morbidity than in those without such conditions [14].

Patients’ tendency to use normalizing attributional styles that see depression as a normal consequence of ill health, along with professionals’ conceptualizations of depression as justifiable and difficult to manage, especially in older adults, are possible reasons for the under-detection and problems initiating treatment of depression in primary care [15,16]. Less is known about barriers to detection and management of depression care in people with LTCs. However, barriers to optimal depression care for people with LTCs can partly be explained by the time-limited nature of primary care, in which clinical decision-making is often centered around prioritizing competing patient demands. This is especially true in health settings such as the NHS, where the management of LTCs is driven by guidelines and treatment algorithms that focus on single diseases. In these time-limited and highly structured environments, competing demands on the time of health professionals often lead to prioritization of physical health problems [17,18], and patients are similarly predisposed to focus on their physical rather than mental-health problems [19]. Additionally, a lack of congruence between the conceptual language used for depression by patients and professionals, along with deficits in communication skills on the part of both groups, can lead to uncertainty about the nature of the problem and reduce opportunities to develop appropriate treatment strategies [18].

There has been a growing emphasis on the role of educational and organizational interventions to overcome barriers to managing depression in primary care [20]. However, simple educational strategies that focus on guideline implementation are largely ineffective compared with more complex interventions that combine clinician education with enhanced roles for non-medical specialists (case management), and a greater degree of integration between primary and secondary care (consultation liaison) [21]. In the USA, successful strategies to improve depression outcomes have been informed by the chronic care model, which emphasizes that care improvement is linked to the redesign of interdependent components in health systems, delivery systems, patient and provider relationships, decision support tools, clinical information systems, community resources and organizational factors, such as leadership [22].

The core ingredient of interventions that have drawn on the chronic care model is collaborative care, which typically involves a multiprofessional approach to care, structured management plans, scheduled follow-ups, and enhanced inter-professional communication [23]. Collaborative care is more effective than standard care at improving depression outcomes over both the short and long term [24]. Evidence is also accumulating that collaborative care can improve depression care and outcomes in patients with LTCs such as diabetes [25]. In patients with depression who have poorly controlled diabetes and/or CHD, collaborative care involving nurse case managers working closely with GPs can significantly improve control of both medical disease and depression [26].

Given the overwhelming weight of evidence in favor of collaborative care, there have been calls for future work to focus on translation of research into practice [27,28]. However, it is still relatively uncertain whether collaborative care is cost-effective in non-managed healthcare settings outside of the USA, either for major depressive disorder [29] or for depression and LTCs [30,31]. There is also uncertainty about whether collaborative care can improve access and quality of care for people with depression of varying severity, especially in the presence of LTCs. There is thus scope for research that addresses whether collaborative care models can be adapted to structure and organize the care of co-morbid depression of varying severity (including mild to moderate) in people with LTCs. Finally, the cost-effectiveness of collaborative care has largely been assessed in the context of large clinical trials, and long-term benefits may be contingent on the continued presence of research infrastructure. There is only modest evidence that quality improvement programs for depression such as collaborative care can improve the process and outcome of care in more naturalistic, non-academic settings that include patient choice and the usual care providers [32].

This pragmatic trial therefore aims to test the cost-effectiveness of collaborative care for depression of varying severity in people with diabetes and/or CHD in primary-care settings that resemble naturalistic conditions that include patient choice and the delivery of low-intensity mental-health services by usual care providers (that is, IAPT).

## Methods

### Background to the trial

The COINCIDE trial was designed by a multidisciplinary team at the University of Manchester as part of the Greater Manchester (GM) Collaboration for Leadership Applied Health Research and Care (CLAHRC) funded by the UK National Institute for Health Research (NIHR). The GM CLAHRC is one of nine CLAHRCs established by the NIHR in 2008. CLAHRCs are partnerships between universities and their surrounding NHS organizations (including primary care) with a strategic focus on evaluating new therapeutic and organizational approaches to chronic disease management, and to support more rapid translation, adoption, and diffusion of research findings into improved outcomes for patients across wide geographical areas. In this sense, CLAHRCs are an innovative model to fill the second translational gap by identifying and evaluating interventions that are effective and appropriate for routine use in the NHS [33].

The twin goals of the GM CLAHRC are to improve quality of care and support for patient self-management and to reduce inequalities in health and access to care for people with vascular disease. Its reach is extensive, covering inner-city, suburban, and semi-rural districts with diverse populations, including deprived and affluent communities, and areas with high and low densities of black and minority ethnic (BME) groups. The program also supports activity in implementation science to translate research findings into practice. This focus on feasibility and implementation in part underpins the pragmatic approach adopted in our trial. Compared with explanatory trials, which impose rigorous restrictions on study entry and treatment delivery to maximize internal validity, pragmatic trials look to answer questions about the overall effectiveness of interventions using populations, settings, and interventions that resemble those found in routine care. In this sense, pragmatic trials are best placed to generate data that can be generalized to other contexts and offer opportunities to evaluate cost-effectiveness.

This study will be a pragmatic cluster randomized controlled trial (RCT) of effectiveness and cost-effectiveness of a collaborative-care intervention for improving access and quality of care for depression in people with diabetes and/or CHD compared with usual primary care in the UK NHS.

### Study design

**Figure 1:**
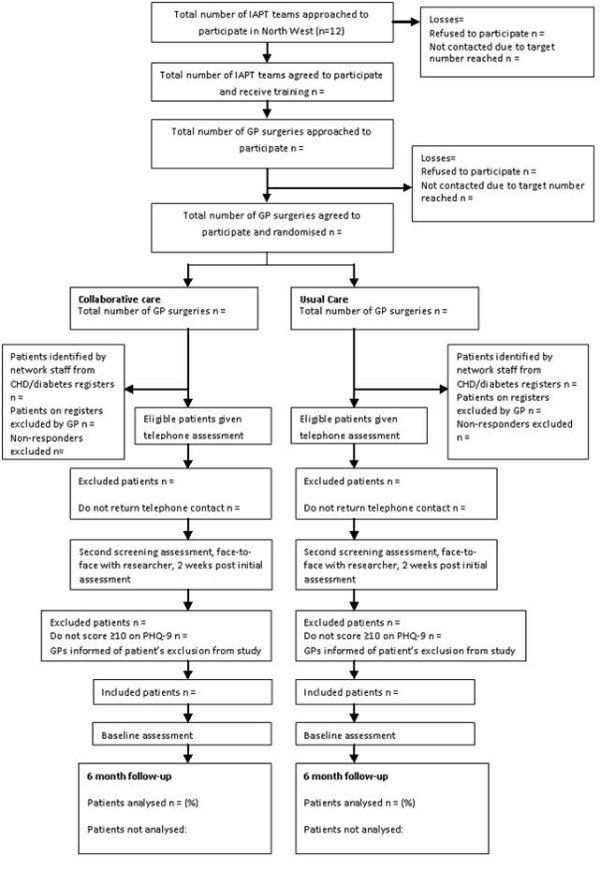
**CONSORT diagram for the COINCIDE study. **The patients are divided into the collaborative care (experimental) and the usual primary care (control) groups.

A previous platform trial of collaborative care that combined individual and cluster randomization showed that the effects of the intervention were partly mediated by organizational factors [34]. COINCIDE will therefore use a cluster design to protect against bias caused by contamination and type II errors. Clusters (general practices) will be allocated to two alternative arms (Figure 1).

Collaborative care is a complex intervention that draws on the principles of the chronic care model and typically encompasses a range of organizational, professional, and patient-level interventions. First elaborated on by Katon and colleagues at the University of Washington to describe mental-health interventions delivered by a patient’s GP and a psychiatrist [35], collaborative care has since undergone evolution leading to an intervention involving [36]:

• A multiprofessional approach to patient care delivered by a GP and at least one other health professional (for example, a nurse, psychologist, psychiatrist, or pharmacist);

• A structured patient management plan that facilitates delivery of evidence-based interventions (either pharmacological or non-pharmacological);

• Scheduled patient follow-ups on one or more occasion, either in person or by remote communication (for example, telephone);

• Enhanced inter-professional communication between the multiprofessional team who share responsibility for the care of the depressed patient (for example, team meetings, case conferences, supervision).

A key component of collaborative care is the introduction of a care (or case) manager in primary care. The case manager acts as the conduit between patients and professionals in primary and specialist care, and works as the patient’s supporter to jointly determine problems, set goals, and action plans, and to offer education and problem-solving skills as ways to promote better patient self-care.

Case managers can be employed from a variety of professional backgrounds. In the USA, registered nurses employed by privately insured primary-care cooperatives have worked as case managers in trials of collaborative care for people with depression and medical co-morbidity. This model of case management is feasible in settings where nurses are already highly skilled in the medical management of people with chronic disease and where there is capacity to integrate other elements of collaborative care with the delivery of routine primary care. A similar, potentially sustainable model of nurse-led collaborative care is being piloted in Australian primary care for patients with depression and heart disease or diabetes [31]. In the UK NHS, practice nurses may also be well placed to case-manage patients with depression and LTC. Practice nurses are central to routine chronic disease management, and patients are accustomed to nurse-led clinics in primary care. Additionally, there is some evidence that patients perceive nurses as more holistic and approachable than other health practitioners, and afford more time to interactional tasks that emphasize patient-centered healthcare [37].

However, in the UK, primary-care consultations are time-limited, and clinical activity is highly circumscribed by the need to meet quality improvement targets set by the QOF, thereby reducing the opportunities for nurses to devote time to training and additional tasks associated with the collaborative care of patients with depression and LTCs [18]. Additionally, because practice-nurse consultations are very structured and driven by guidelines and templates, it might be harder to train nurses to take on additional duties and new ways of working [38,39]. For example, when promoting self-care for patients with diabetes, primary-care nurses are familiar with traditional didactic health education approaches, but might find it difficult to give lifestyle counseling that draws on motivational and behavioral approaches typically used by mental-health professionals [40]. Moreover, training primary-care professionals to improve recognition and outcome of primary-care depression has not proven a success in UK settings, indicating a need to test consultation liaison models such as collaborative care to improve major depressive disorder in some patients [41].

In the COINCIDE trial, the case managers will be psychological well-being practitioners (PWPs) who are graduate psychologists who provide high-volume, low-intensity psychological interventions based on a cognitive and behavioral model for patients with depression and anxiety disorders. PWPs are employed by IAPT teams, who are commissioned in each primary-care trust (PCT). IAPT was established in 2006 to improve access to routine, evidence-based, first-line treatment for adults of working age following calls from Layard [42] for a new deal for people with depression and anxiety disorders. As part of the UK government’s commitment to mainstream mental health in England and to reduce inequities between physical and mental-health services [43], the scope of IAPT will be broadened to include a standalone program for children and young adults, and the development of care models for people with LTCs, medically unexplained symptoms, and severe mental illness [44].

Training PWPs to become case managers to deliver collaborative care for people with LTCs and depression thus not only fits the strategic goals of the NHS and IAPT, but also meets the needs of the CLAHRC to identify and evaluate interventions that stand the greatest chance of implementation in routine settings. As well as being premised on strategic and pragmatic goals, the decision to train PWPs in this trial also draws on a robust evidence base that shows that the effect size of collaborative care is higher in studies in which case managers have a specific mental-health background [24].

### Study setting and sample size

#### Recruitment of IAPT services

This study will be based in primary care across a geographical area that includes 13 PCTs in the north west of England. We will recruit IAPT services that provide low-intensity psychological therapies. All IAPT teams that fall within the catchment area of the target PCTs will be eligible for inclusion. Within each IAPT service, clinical team leaders will nominate PWPs to receive the training in the new collaborative-care model for people with depression and diabetes/CHD. The ratio of trained to untrained PWPs in each IAPT service will vary depending on the size of the team; however, it is estimated that we will need to train up to two PWPs per IAPT team to accommodate the case loads generated by the clinical trial. Furthermore, in the context of this pragmatic trial, training multiple therapists protects the trial from drop-out from PWPs, and allows for more robust assessment of therapists’ effects. Each IAPT team that is included in the trial will retain capacity to offer psychological therapy services to other groups, including patients referred from general practices that are randomized to the usual care arm.

#### Recruitment and randomization of GP practices

We will recruit 30 general practices, representing approximately 5% of the total number of practices from the target areas in the northwest of England. All general practices that fall within the catchment area of the trial will be eligible for inclusion. Practices will be allocated to collaborative care or usual care using a central randomization service provided by the Clinical Trials Unit at the Christie NHS Foundation Trust, Manchester, UK. Randomization will be by minimization based on practice size and area deprivation using the Index of Multiple Deprivation [45]. In addition, we will target areas of the northwest (for example, East Lancashire) that have a high density of people from BME groups, especially South Asians. This policy will allow us to enroll sufficient numbers of patients of South Asian origin to pilot-test the acceptability of the intervention in this group. In this trial, participants of South Asian origin are defined as people born in India, Pakistan, Bangladesh and Sri Lanka and their descendants, but excluding those born (or descended from those born) in Nepal, Bhutan, the Maldives, Tibet, Afghanistan, or the Islamic Republic of Iran [46].

Recruitment and training will be undertaken in two sequential waves. This strategy will allow more flexibility when recruiting IAPT and general practices and offers opportunities to: 1) stagger recruitment of IAPT teams and general practices, and 2) run two separate rounds of case-finding, thereby concentrating data collection around fixed periods in the trial calendar, leading to more feasible project management.

### Patient recruitment

#### Sample size

Based on a UK trial of collaborative care for major depressive disorder [29] we have powered this trial to detect a standardized effect size of 0.4. If each of the 30 practices (15 in each arm) recruits 15 patients at baseline (450 in total), the study will have > 80% (81.2%) power to detect this size of effect at the 5% level of significance, allowing for an intraclass correlation coefficient (ICC) of 0.06 (that was identified in a previous Phase II trial of collaborative care) and a 20% attrition rate.

#### Inclusion criteria

GP practices will be eligible for inclusion if they hold and maintain a QOF register of: 1) patients with coronary heart disease (QOF indicator CHD1: secondary prevention of coronary heart disease) [47]; and 2) all patients aged 17 years and over with diabetes mellitus, which specifies whether the patient has type 1 or type 2 (QOF indicator DM19: diabetes mellitus) [47].

The QOF does not specify how the diagnosis of diabetes/CHD is to be made. For the purposes of the QOF, a record of the diagnosis is sufficient as evidence of diabetes/CHD. The CHD register will include all patients who have had coronary artery revascularization procedures such as coronary artery bypass grafting and those with a history of myocardial infarction, but does not generally include patients with cardiac syndrome X.

The QOF recommends separate coding of type 1 and type 2 diabetes. Where paper or electronic records fail to distinguish between the two conditions, the QOF register codes people diagnosed before the age of 30 years and requiring insulin within 1 year of diagnosis as having type 1; all other cases are coded type 2. The diabetes register generally excludes patients under 16 years and women with gestational diabetes.

Patients of 18 years or over attending each practice will be eligible for inclusion if: 1) they are listed on the QOF registers held by the practice for CHD and/or type 1 or type 2 diabetes mellitus, and 2) they have persistent depressive symptoms (≥10 on the nine-item Patient Health Questionnaire; PHQ-9) [48].

To enhance the representatives of the sample and in keeping with the pragmatic design, we will include patients with diabetes and/or CHD who are already receiving anti-depressant medication but still have score of greater than or equal to 10 on the PHQ-9 at the initial telephone assessment. Patients who are not currently engaged in psychotherapy but have received psychotherapy in the past will be eligible to join the study. Unlike previous large trials of collaborative care, we will also include non-English speaking patients of South Asian origin whose first language is Gujarati, Urdu, or Bengali; these languages comprise the most commonly used among British South Asians in the northwest of England.

Patients will be excluded if they are aged less than 18 years; they refuse consent; their GP removes them from the practice diabetes/CHD database; they have severe and enduring mental disorders (psychosis, or bipolar I or bipolar II disorder); they have active suicidal thoughts and intent, and require immediate care from a crisis management team; or their use of alcohol or non-prescription drugs requires clinical intervention.

#### Screening and recruitment of patients

Patient recruitment is widely regarded as the most challenging aspect of conducting both individual-patient and cluster RCTs [49]. Failure to recruit target sample sizes can lead to costly and lengthy extensions and loss of power. In primary care, time constraints, GPs forgetting to recruit, and identification of too few eligible patients threaten recruitment rates [50]. Furthermore, cluster trials in health settings are at high risk of selection bias because of selective recruitment of patients by professionals who are aware of intervention allocation [51], and through systematic differences in referral behavior between the clusters in each arm [34].

To ensure adequate recruitment of patients and to minimize the risk of selection bias, we will recruit patients by case-finding of eligible patients with depression and CHD/diabetes rather than relying on direct GP referral. Such reliance in mental-health trials has proven to be too slow and inefficient [52], and recent individual-patient and cluster trials of collaborative care in the UK and USA have shown that screening eligible patients for depression is an efficient and acceptable way to improve recruitment rates. Accordingly, in partnership with the NIHR Mental Health Research Network (MHRN), the practice staff will work with clinical studies officers (CSOs) to compile a mailing database of all patients on the diabetes/CHD registers held in each participating general practice. GPs in participating practices will then be paid to review this database to exclude patients that do not meet the trial inclusion criteria and to remove recently deceased patients. CSOs will then assign study identification numbers to each patient on the mailing database, and in partnership with the CLAHRC team, will send a study pack to all eligible patients on the database, including a study invitation letter, an information sheet, a consent form, and a request for translation. Reminder letters and replacement study packs will be sent 3 weeks later to patients who have not responded to the initial mailing. Because mail-based recruitment is known to yield poor returns, non-responders to the reminder letter will also be telephoned by CSOs [53]. Non-responders of South Asian origin and those do not speak English will be telephoned by a member of the COINCIDE study team proficient in Bengali, Gujarati, and Urdu, who is blinded to intervention allocation.

After receipt of consent forms, CLAHRC research staff blinded to the intervention allocation will make an initial screening appointment with eligible patients. Screening will be undertaken over the telephone using the PHQ-9. Patients who score greater than or equal to 10 on the PHQ-9 will be informed that they provisionally meet the inclusion criteria for entry into the trial; patients who score less than this will be informed that they are not eligible for the study.

Scores greater than or equal to 10 on the PHQ-9 are indicative of major depression [48]. Although using a more conservative cut-off of greater than or equal to 12 might improve the accuracy of predicting major depression with the PHQ-9 [54], using the lower cut-off of 10 has two advantages. First, we will be able to make comparisons with international studies that have used similar thresholds for depression. Second, using the lower cut-off of greater than or equal to 10 allows detection of sub-threshold depression or ‘diabetes -elated distress’, which has been identified as a key mediating factor between depression and glycemic control in diabetes [55]. It could be argued that such levels of distress should be recognized earlier and managed in people with diabetes.

Administration of the PHQ-9 in person or by telephone produce similar results, making telephone assessments a reliable screening method for depression in primary care [56]. Furthermore, the case-finding ability of the PHQ-9 is comparable with that of standardized diagnostic clinical interviews when used in medical settings [57], and is as good as, if not superior to, the Hospital Anxiety and Depression Scale when used to screen for depression in non-psychiatric populations [58].

Remission is common for mild to moderate depression, and the ability of this trial to detect treatment effects will be improved if the sample includes only patients with persistent depressive symptoms. To exclude patients with transient and spontaneously remitting depression research staff blinded to the intervention allocation will therefore visit patients who scored greater than or equal to 10 on the initial telephone PHQ-9 screen to undertake a second PHQ-9 screen. Patients who score greater than or equal to 10 at this second PHQ screening appointment will then be invited to complete baseline assessments and enter the trial. The researcher will tell the participant’s GP about their involvement in the study. Patients who score less than or equal to 9 at this second screening appointment will be informed that they do not meet the study inclusion criteria and will be excluded; their GP will be informed by the research team that they will take no further part in the study at this stage. A CONSORT flow diagram of the recruitment and screening strategy is shown in Figure 1.

Researchers blinded to the intervention allocation will forward the details of those patients who are eligible to join the trial (those who scored ≥10 on PHQ-9 at the second screening appointment) to the COINCIDE trial manager. The trial manager will enter these details onto a password-protected and encrypted database. Within 3 working days of the second screening appointment, the trial manager will contact the clinical lead of the IAPT team to pass on contact details of patients in the collaborative-care arm who have met the inclusion criteria. PWPs will then contact the patient directly to set up a first appointment.

### Intervention design

#### Collaborative care

PWPs will case-manage people with diabetes and/or CHD and depression in partnership with the patient’s GP and a practice nurse. Each patient will receive up to eight sessions with the PWP over 12 weeks. Sessions can be conducted in person or by telephone, depending on patient preference. The first session will be 45 to 60 minutes long, during which the case manager will complete a detailed biopsychosocial patient assessment; elicit the patient’s ABC-E (autonomic, behavior, cognition, environment)) model about diabetes and/or CHD and links with low mood; briefly educate the patient about diabetes/CHD and depression; assess risk; and introduce the treatment manual and develop with the patient a main problem statement. All other sessions will be between 30 and 40 minutes in length. During the second session, patients will select which intervention they prefer, and active treatment will start during the third session. Although outcomes in depression scores are the main focus of this study, treatment manuals have been designed to incorporate interventions that address any symptoms of anxiety that may present alongside depression. This assessment and treatment schedule is consistent with the delivery of brief psychological therapies in the NHS, where six to eight sessions is a common treatment length.

A 10 minute collaborative meeting (by telephone or in person) between the patient and the case manager and a practice nurse will take place at the end of the second and eighth sessions. These collaborative meetings will focus on reviewing patients’ progress with their main problem statement and goals, reviewing relevant health outcomes (for example, HbA1_c_, lipids), and a medication review. The collaborative meeting at the end of the eighth session also includes opportunities for the collaborative-care team to discuss with the patient the next steps to be taken, either to maintain health gains or, if the patient’s mood has not improved, to review medication (for example, change anti-depressant or increase dose) or referral to higher-intensity interventions. The rationale for these collaborative meetings is to embed collaborative ways of working into the care of patients with diabetes/CHD beyond the initial 3-month treatment phase, especially in relation to goal-setting and action planning.

The key principles of the intervention are that it is patient centered and includes partnership working, proactive follow-up, and integrated communication and care between the patient, a mental-health professional (PWP), and a GP and/or a practice nurse. The type of psychological intervention used will be chosen by the patient and will be goal-orientated to primarily improve mental heath outcomes. However, the delivery of depression treatments in this population might also support the management of patients’ physical health, for example, by giving lifestyle advice about diet and exercise. In this sense, the COINCIDE trial aims to use collaborative care as a platform to integrate physical and mental health care for people with depression and diabetes and/or CHD.

#### Usual care

Patients allocated to the control group will receive their usual care by their primary-care team. For patients with diabetes and CHD, usual care includes meeting QOF clinical indicators as part of the General Medical Services and Personal Medical Services [47]. Because this is a pragmatic trial, patients in the usual care group will still be eligible to receive anti-depressant treatment and referral for psychological therapy. However, if patients in the usual care group are referred to an IAPT team for psychological therapy they will not receive treatment from a PWP who has been trained in LTCs as part of the COINCIDE trial. It is not yet known if collaborative care delivered in the UK NHS as part of a low-intensity (or step 2) intervention is any more effective than standard care for depression in people with LTCs. In this sense, patients in the usual care group with recognized depression are not disadvantaged, as they will still have access to evidence-based treatments.

Additionally, we do not anticipate that participation in the trial will radically alter care pathways for depression in the control group. There is solid evidence that case-finding or screening for depression does not in itself lead to improvements in detection and management of depression by clinicians [59], nor is case-finding for depression known to be a necessary component of effective collaborative care interventions for depression. When used as a mechanism to increase recruitment in a clinical trial, screening alone is unlikely to affect the quality of care available to patients with recognized depression, thus preserving the comparison in the trial between collaborative care and usual care for depression. We will record all aspects of the patients’ usual care in the control group, and the follow-up assessments will be identical to those used in the intervention group.

#### Stepped-care algorithm and risk management

An adapted stepped-care approach will be used based on NICE recommendations for treating depression [9,10]. Where demand exceeds supply, stepped-care models potentially offer a framework to organize and provide services in the most efficient and effective way by supporting patients, carers, and practitioners in identifying and accessing the least restrictive (and least intensive) but most effective intervention first [20]. If a patient does not benefit from the intervention initially offered, or declines an intervention, they should be offered an appropriate intervention from the next step. In theory, the stepped-care model should be self-correcting, because patients can be stepped up when they are not progressing to agreed targets. Currently, collaborative care is recommended as a step 3 or high-intensity intervention for depression in cases where the patient also has a LTC and associated functional impairments. However, in keeping with previous studies of collaborative care [25], the COINCIDE trial defines collaborative care as a health-services intervention to improve the quality and choice of evidence-based care for depression, rather than as a therapeutic intervention in itself. In this sense, collaborative care is an organizational framework that can facilitate and improve the delivery of all types of therapeutic interventions.

Following formal review after the fourth and eighth sessions, if patients have persistent depressive symptoms (<50% decrease in severity on PHQ-9) then PWPs, in partnership with the patient and in consultation with their clinical supervisor, will discuss the most appropriate action(s) from a range of options: 1) change low-intensity psychological intervention; 2) if patient is not on antidepressants, ask patient to see GP to discuss this option; 3) if patient is already on antidepressants then ask patient to see GP to discuss increase in dose or change of anti-depressant medication; 4) step up to a higher-intensity intervention (which will usually be cognitive and behavioral therapy; CBT). Weekly monitoring between the second and eighth session using tools from the IAPT minimum dataset will support PWPs in reviewing the patients’ progress and implementing the stepped-care algorithm. The local collaborative team (PWPs, supervisors, and GPs) will be able to consult the trial clinical team (LG, KL, CCG) about starting, or adjusting dose, or changing anti-depressant medication, as we recognize that specific medication expertise may not be available within the IAPT team.

Where there is concern about risk of self-harm, suicide, neglect, or harm to others, patients will be stepped up to community mental-health services for assessment and management. This decision will be made in collaboration with the local supervisor, with additional support from the trial clinical team (LG, KL, CCG) where required.

### Intervention components

#### Guided self-help

The treatment, partly delivered with the aid of a patient manual and workbook, is based on an individual-guided self-help program for people with depression and diabetes/CHD delivered by PWPs in collaboration with a GP and practice nurse. Guided self-help based on the principles of CBT is one of a range of low-intensity psychological interventions recommended by NICE [9,10] for people with persistent sub-threshold depressive symptoms and mild to moderate depression and should include the provision of written materials of an appropriate reading age (or alternative media to support access); be supported by a trained practitioner, who typically facilitates the self-help program and reviews progress and outcome; and consist of up to six to eight sessions (in person or by telephone), normally taking place over 9 to 12 weeks, including follow-up.

In the COINCIDE trial, patients in the collaborative-care arm will be offered a choice of brief psychological interventions: behavioral activation (BA), graded exposure, cognitive restructuring, and/or lifestyle changes. Medication management will also be used to ensure that patients have the necessary knowledge to make an informed choice about whether to start an anti-depressant.

#### Behavioral activation

BA is premised on the notion that depression is functional, whereby depressive feelings and thoughts stimulate avoidant behavior, leading to negative reinforcement and a reduction in positive reinforcement and in normal (routine, pleasurable, and necessary) activities. This behavioral model of depression acknowledges that the experience of being depressed includes bi-directional relationships between life events and modified reinforcement schedules and secondary problems such as avoidant coping. The format adopted in this trial is a four-step BA plan to support patients to reschedule activities to reintroduce positive reinforcement, improve thoughts and mood, and reduce avoidant behaviors. In partnership with PWPs, patients will draw up and self-monitor activity schedules, and identify goal-oriented behaviors likely to reinforce anti-depressant environmental contingencies.

BA is as effective and acceptable as higher-intensity CBT for the treatment of depression [60]. Moreover, because it adopts a more parsimonious therapeutic approach than CBT, it can potentially be delivered by low-intensity therapists (such as PWPs), leading to opportunities for implementation in routine settings [61].

#### Graded exposure

The initial stages of the intervention involve the PWPs educating patients about the effects of avoidance and the role of safety behaviors in the maintenance of anxiety, which can in turn lead to withdrawal and depression. Using the patient’s individual symptoms, cognitions and behaviors, the PWPs illustrate how vicious circles (symptom → catastrophic thought → fear → avoidance → disability → symptom) maintain the problem.

Patients are then supported in session to draw up a graded hierarchy of fear-eliciting situations. These take the form of a series of behavioral experiments, carried out between sessions, during which irrational expectations are challenged. Patients are encouraged to work up the hierarchy, remaining in these fearful situations until anxiety has significantly decreased. This process must be completed several times until minimal anxiety is experienced before the patient proceeds to the next step on their hierarchy. A successful exposure intervention results in a reduction in the anxiety response and increased behavioral tolerance of identified triggers. In cases where the PWP identifies specific anxious cognitions within the maintenance cycle that are resistant to exposure, the patient will be helped to consider the evidence for and against these fears and, where appropriate, carry out behavioral experiments between sessions, during which anxious predictions are examined and reflected upon. The successful addition of this intervention should result in a cognitive shift, whereby the patient no longer negatively misinterprets physical symptoms and no longer engages in avoidance or unhelpful safety behaviors. Where anxiety symptoms are longstanding, or complex or specific phobias directly affect self-management, PWPs will facilitate stepping up to high-intensity CBT.

#### Cognitive restructuring

Cognitive restructuring is used to identify and challenge seemingly rational and believable negative thoughts by reframing them as more accurate and realistic beliefs. In COINCIDE, patients will use thought diaries and engage in behavioral experiments to identify situations that trigger negative, often automatic, thought processes, and will work with the PWP to challenge these negative thoughts with more positive and goal-oriented ones.

#### Lifestyle advice

PWPs will work with patients to help them identify and implement lifestyle changes to lead a healthier lifestyle, including addressing sleep habits, diet, exercise, and relaxation.

#### Medication management

PWPs will work with the patient to assess whether they are taking medication as prescribed, address any worries and concerns about the medication and side-effects, provide good-quality information about medication to address concerns, and help to arrange a review of medication with the GP if this is indicated because of patient and/or therapist concerns about side-effects and/or failure to improve.

### Training and supervision

#### Training and supervision of case managers

Case-management training will be delivered by a multidisciplinary team of high-intensity and low-intensity therapists, academic GPs, and consultant psychiatrists, along with disease specialists in diabetes and CHD. PWPs will receive an initial 1-week (5-day) training course that includes classroom sessions and interactive skills practice, along with video demonstrations of conducting a biopsychosocial patient assessment, formulating a shared problem statement, and the collaborative team meeting. PWPs will use a training manual during the training week (Table 1).

**Table 1:** Description and aims of the training

**Day**	**Section**	**Aim**
1	Introduction to collaborative care and LTCs – an overview of the evidence base	Understanding diabetes and CHD
2	Patient-centered interviewing and promoting change	Developing shared problem statements and goal-setting
3	Introduction to psychological interventions for depression and LTCs	Medication management and lifestyle interventions
4	Delivering behavioral activation and cognitive restructuring for depression and LTCs	
5	Maintaining health, effective liaison, supervision and monitoring	

Case managers will receive 1 hour of individual supervision on a weekly basis from a senior mental-health practitioner within their IAPT team; this may be undertaken in person or by telephone, depending on case manager and supervisor preference and availability. This supervision will be structured in accordance with the IAPT PWP case-management supervision guidelines [62,63]. Using this approach, at each session new cases, cases with risk, those not improving by the eighth session and those with missed sessions (did not attend; DNA) are routinely discussed; in addition, every case is reviewed every 4 weeks. The case manager will report the case and include the problem statement, questionnaire scores (PHQ-9; Generalized Anxiety Disorder Assessment-7 [GAD-7]) and risk issues, medication, goals and treatment plan, with the addition of items that specifically focus on the patient’s LTC: the effect of their depression on their physical health and self-management, treatment concordance, progress with diabetes and/or CHD outcomes (for example, hemoglobin A1_c_, lipids), and any additional case-management issues. In addition, case managers will have fortnightly access to the equivalent of 1 hour of clinical skills supervision, conducted in groups and facilitated by a senior mental-health practitioner within their IAPT team, allowing the opportunity for in-depth discussion and further skill development arising from specific cases.

PWPs will keep and maintain notes for the patients they treat using the GP practice computer systems, if this is acceptable to GPs and practice managers. In addition, as part of reporting for the trial, PWPs will keep a monitoring sheet for each patient. This monitoring sheet reports how many sessions patients attended on average, the delivery mode in which they received follow-up (in person or by telephone), and what interventions patients selected.

#### Train the trainer sessions

After completion of follow-ups, irrespective of outcome, the COINCIDE training team will offer training in the collaborative-care model to previously untrained supervisors and clinical leads from IAPT teams enrolled in the trial. These ‘train the trainer’ sessions will be given so that IAPT teams can draw on local expertise to train and then supervise additional step 2 PWPs in using collaborative care for treating depression and diabetes/CHD. This approach will support IAPT teams to build capacity to meet demand for low-intensity psychological interventions from people with diabetes/CHD. This strategy also partly meets IAPT objectives to roll out psychological therapy services to people with LTCs [44], and is consistent with the GM CLAHRCs commitment to support implementation of research into practice.

#### Training workshop for practice nurses

A separate half-day workshop will be run for practice nurses drawn from GP practices randomized to the collaborative-care group. This workshop will be shared with PWPs trained in the collaborative-care model and will focus on delivering integrated care and effective consultation liaison.

### Outcome assessment

#### Primary outcome

The mental-health needs of people with LTCs have historically been under-recognized and under-served in primary care, and the main aim of this trial is to improve the access to and quality of depression care for people with two exemplar LTCs: diabetes and/or CHD. The interventions used are designed primarily to improve depression rather than both depression and physical health outcomes. The primary outcome in this trial is therefore change in depressive symptoms. However, using low-intensity psychological interventions, including lifestyle counseling, to treat depression may also be beneficial for diabetes/CHD, leading to opportunities for therapeutic synergies across multiple conditions. Accordingly, we will also test the effects of collaborative-care interventions on a range of secondary outcomes related to diabetes and CHD and self-care.

The 90-item Symptoms Checklist (SCL-90) depression scale will be used to assess changes in severity of depressive symptoms at 6-months [64]. Response to treatment is defined as greater than or equal to 40% reduction in SCL-90 scores [65]. We will also use the PHQ-9 at baseline and follow-up to measure changes in dichotomous diagnosis of depression [48].

#### Secondary outcomes

A number of secondary outcomes will be measured, using various instruments.

• The five-item EuroQoL (EQ-5D) is a standardized generic measure of health-related quality of life (HRQOL), which is suitable for use in people with a wide range of health conditions, and is recommended by NICE for economic evaluations in clinical trials. It can be completed by patients, and may be used as a postal questionnaire or in interview [66]. Further, it has proven reliability, validity, and responsiveness in type 2 diabetes, suggesting that it can be used for modeling health outcomes in economic evaluations of interventions for people with type 2 diabetes [67].

• The World Health Organization Quality of Life brief measure (WHOQoL-BREF) is a measure of global QOL, which has been validated in a large international population for a wide range of illnesses [68]. The brief measure is a 26-item version of the 100-item WHOQoL measure. QOL is measured across four domains that have been shown to be salient: physical, psychological, social, and environmental. Items are measured using a five-point Likert-type response; a low score for each item is indicative of poor QOL. Three items are negatively phrased and reverse scored. Scores from each domain are calculated using the mean score for the items within that domain, and multiplying the mean score by four, so that scores taken from the WHOQoL-BREF may be compared and contrasted with the 100-item WHOQoL measure. Additionally, the WHOQoL contains a single-item scale for QOL. The first item on the questionnaire ‘How would you rate your quality of life?’ can be interpreted individually as a measure of patient-reported QOL. Additionally the total score of the WHOQoL has been shown to operate effectively as a unidimensional measure of QOL [69].

• The Diabetes Quality of Life (DQoL) tool measures both the physical and emotional effects of diabetes and diabetes treatment using five scales: satisfaction with treatment; impact of treatment; worry about the future effects of diabetes; worry about social and vocational issues; and overall well-being [70].

• The Seattle Angina Questionnaire (SAQ) is a reliable and predictive tool that measures five clinically important dimensions of functional status in people with coronary artery disease [71].

• The seven-item Generalized Anxiety Disorder (GAD-7) is a self-report scale measuring symptom severity of GAD over the previous 2 weeks on a four-point Likert scale with a cut-off of greater than or equal to 8 [72].

• Self-efficacy: changes in self-efficacy cognitions (that is, a person’s confidence to produce a desired outcome) are theorized to lead directly to changes in self-rated health and thereby influence healthcare utilization. Validated scales used in the evaluation of the Expert Patient Program will be used [73].

• Patient self-management behavior will be evaluated using the Health Education Impact Questionnaire (heiQ). This 42-item scale measures eight independent dimensions, including positive and active engagement in life, health-directed behaviors; skill and technique acquisition; constructive attitudes and approaches; self-monitoring and insight; health-service navigation; social integration and support; and emotional well-being. The heiQ has shown preliminary evidence of construct validity [74].

• The Client Satisfaction Questionnaire (CSQ-8) is an eight-item self-administered questionnaire collected at the end of service delivery and scored using a four-point Likert scale. The CSQ-8 scores range from 8 to 32, with higher values indicating higher satisfaction. Scores are correlated with change in self-reported symptoms as measured by the Client Checklist, a self-report symptom checklist made up of items from the SCL-90. The CSQ-8 gives equivalent results to the CSQ-18, and is shorter and quicker to complete, lending it a practical advantage for use in primary care [75].

• Healthcare utilization will be examined using the Patient Service Utilization questionnaire [29].

• The Patient Assessment of Chronic Illness Care (PACIC-5A) is a patient-centered assessment of implementation of the chronic care model (CCM), which focuses on the receipt of patient-centered care and self-management behavior [76]. In patients with diabetes, the PACIC was shown to be associated with increased exercise and receipt of appropriate laboratory assessments and self-management counseling [77]. PACIC assessments of the implementation of CCM have also been shown to be independently associated with improved self-management behaviors and patient-centered outcomes in adults with a wide range of LTCs, including CHD [78]. The five subscales of the PACIC are patient activation, delivery system/practice design, goal-setting/tailoring, problem-solving/contextual, and follow-up/co-ordination. Six additional items measure minimal contact behavioral counseling interventions provided in primary care, in accordance with the ‘Five As’ construct (assess, advise, agree, assist and arrange) devised by the Canadian Task Force on Preventive Health Care and the US Public Health Service [79,80].

• The ENRICHD Social Support Instrument (ESSI) is a brief seven-item measure of perceived social support[81]. The scale contains six social support items that have been shown to be individually predictive of poor outcome in patients with cardiac disease, and one item that assesses partner status. The scale has acceptable reliability and validity.

• The Sheehan Disability Scale [82] is a brief measure of disability in carrying out life activities across the three domains of work, social, and family life.

• The Relationship Scales Questionnaire (RSQ) is a measure of attachment styles containing 30 short statements describing interpersonal characteristics in close relationships to be rated on five-point Likert scale. Each statement contributes to secure, dismissing, preoccupied, or fearful attachment patterns. Scores for each attachment pattern are derived by taking the mean of items representing each attachment prototype [83].

Where no validated and translated version exists for use with South Asian patients who do not speak English, we will culturally adapt and translate all outcome assessments [84]. The trial will employ a South Asian language specialist who will have responsibility for supporting data collection from Urdu, Bengali, and Gujarati speakers.

### Process evaluation

In addition to primary and secondary outcome measures, we will include measures of characteristics and processes that may predict whether depression and medical outcomes improve within particular groups of patients. Evaluation of predictors of treatment response will include quantitative assessment of the following.

• Patient demographic characteristics, including age, gender, ethnicity, and socioeconomic status. These will be derived from the items used in the General Practice Assessment Questionnaire [85].

• Patient clinical characteristics, including number, type, and burden of LTCs assessed by a self-reported measure of disease burden [86].

• Illness representations, using a modified version of the Brief Illness Perceptions Questionnaire [87], for use in people with multiple morbidities.

• Adherence to treatment intervention with PWP (from contact sheet completed by primary-care practitioner).

• Contextual practice variables: anti-depressant prescription rates, and availability and referral to other mental-health services.

### Measure of contamination

It is possible that PWPs trained by CLAHRC might share knowledge and training about collaborative care with colleagues not enrolled in the COINCIDE trial, thereby subverting the study protocol and threatening the internal validity of the study. However, non-CLAHRC trained PWPs will not be using collaborative care for the duration of the trial when they encounter patients with depression and diabetes/CHD. Thus, the effects of contamination caused by knowledge sharing between PWPs are likely to be minimal. We will measure the effect of contamination between CLAHRC-trained and non-CLAHRC-trained PWPs by assessing changes in knowledge, attitudes, and clinical practice between baseline and follow-up, using previously validated approaches to evaluate depression training programs [88].

The schedule of assessments is shown in Table 2.

**Table 2:** Schedule of assessments

**Patient assessments**	**Time point**		
	**Screening**	**Baseline**	**6 months**
PHQ-9	X	X	X
Demographic variables, past medical and psychiatric history		X	
Current physical illness details (including medication)		X	
Number and burden of diseases		X	
RSQ		X	
Self-efficacy measure		X	X
SCL-90 depression		X	X
EQ-5D		X	X
GAD-7		X	X
DQoL		X	X
SAQ		X	X
WHOQoL-BREF		X	X
heiQ		X	X
ESSI		X	X
AIPQ		X	X
SDS		X	X
Anti-depressant prescription rate and referral to other MHS			X
Healthcare utilization (purpose-designed questionnaire)			X
PACIC			X
CSQ			X
Practitioner assessment			
Change in KAP		X	X

Abbreviations: AIPQ, Adapted Illness Perceptions Questionnaire; CSQ, Client Satisfaction Questionnaire; DQoL, Diabetes Quality of Life; ESSI, Enhancing Recovery in Coronary Heart Disease (ENRICHD) Social Support Instrument; EQ-5D, the five-domain EuroQoL quality of life questionnaire; GAD-7, the seven-item Generalized Anxiety Disorder questionnaire; heiQ, Health Education Impact Questionnaire; KAP, Change in Knowledge, Attitudes, Practice; MHS, mental-health service; PACIC, Patient Assessment of Chronic Illness Care; PHQ-9, the nine-item Patient Health Questionnaire; RSQ, Relationship Scales Questionnaire; SAQ, Seattle Angina Questionnaire; SCL-90, the 90-item Symptoms Checklist; SDS, The Sheehan Disability Scale; WHOQoL-BREF, World Health Organization Quality of Life brief measure.

#### Qualitative process evaluation

Although a considerable evidence base exists for the role of collaborative care in improving treatment of depression [89], there is a recognized gap between the efficacy demonstrated in trials and the implementation of the intervention in everyday practice [28], with particular uncertainty around whether the model will effectively translate to UK healthcare systems [36]. The UK Medical Research Council has highlighted the need for process evaluations to understand the problems of integrating interventions into healthcare settings. Gask *et al*. [89], in an evaluation of collaborative care for depression in primary care in the UK, demonstrated the utility of post-trial interviews of participants, which can provide additional insight beyond pre-trial expectations of performance.

We will therefore undertake a separate qualitative process evaluation to evaluate the extent to which the collaborative-care model was implemented in the intervention GP practices and IAPT teams. The aims of this arm of the process evaluation are to explore: the feasibility and acceptability of collaborative care as experienced by both patients and professionals, with a particular focus on integration of physical and mental health care; the sustainability of collaborative-care models beyond the trial; and the feasibility and acceptability of implementation.

### Professional experiences of delivering collaborative care for depression in long-term conditions

Role congruence, perceived patient acceptability, belief about patients’ capabilities, and perceived effectiveness can all have an effect on the attitudes of primary-care clinicians to treatments and screening [90]. In particular, we will explore whether collaborative care affects the attitudes of professionals toward addressing the physical and mental healthcare needs of people with LTCs in more integrated ways. Gunn *et al*. [91] reported that in their study of depression in primary care, GPs were either ‘integrators’ or ‘separators’. Integrators saw physical and mental health as intrinsically linked, whereas separators tended to deal with physical and mental health problems as distinct. This proposed study will examine whether PWPs trained specifically to address depression in LTCs are able to achieve an integrated approach, and whether they perceive the views of patients and other primary care professionals as congruent with this.

### Patient experience of collaborative care for depression in long-term conditions

Patients’ views about their perceptions of integrated services and also the integration of their physical and mental health care will also be explored. Evidence suggests that the professional background of the case manager is key to the success of collaborative care [24], but it is uncertain whether this is in part associated with patient perceptions and expectations about the role of mental-health professionals compared with physical health specialists such as practice nurses. Patient perceptions and observations about the degree of interaction and integration between professionals will also be explored; for example, whether patients perceive that communication between professionals improved, and whether they report that their conditions were managed in more integrated ways through shared determination of treatment goals or by using interventions that address both physical and mental health problems.

### Sustainability beyond the trial

Although collaborative care has been shown to be effective in managing depression, concerns have been raised about how these effects endure beyond the research context [32]. The process evaluation will explore how successfully the collaborative-care model can be embedded within NHS primary care, where psychological therapy services are provided by usual care providers. For example, we will capture provider and user perspectives to address whether GP practices and existing IAPT services have the capacity to continue working collaboratively, and whether PWPs are the most appropriate professional to train as case managers.

The process evaluation will therefore provide data on both the experience of implementation in practice and the resulting perceptions about the likelihood of the model being integrated in routine practice. These aims will be met through semi-structured interviews, which will be analyzed using normalization process theory (NPT).

### Theoretical model: normalization process theory

Process evaluations are rarely guided by theoretical models, and tend to adopt procedural rather than conceptual approaches to intervention complexity [92]. Theoretical models are necessary to understand and predict problems of integration into practice [92]. This is particularly important for addressing the execution and realization of interventions in a pre-existing operational context such as healthcare settings, where novel interventions must fit into deeply embedded professional and organizational systems.

NPT will be used to develop the topic guides for interviews and provide the framework for analysis. NPT is explicitly concerned with the workability and sustainability of complex interventions, focusing on the routinization of intervention components. NPT asks to what extent novel interventions can be assimilated and embedded into routine practice, making it very consistent with the aim of CLAHRC to identify how interventions can be effectively adopted into actual practice. NPT can therefore be used to address both feasibility of implementation, by recognizing which components of implementation may pose particular barriers, and the likelihood of sustainability, by examining the extent to which the intervention can become integrated within everyday practice.

NPT has been successfully used to guide evaluations of implementation of depression care in healthcare settings [89,91], but has to date not been used to examine embedding of depression interventions for patients with chronic illness. NPT states that implementation can be understood through four generative mechanisms:

• Coherence: the meaning of the practice to actors

• Cognitive participation: engagement, individually and collectively, with the practice

• Collective action: interaction with pre-existing or established processes

• Reflexive monitoring: how the practice is assessed and understood by the actors.

These constructs will be used to guide the analysis of qualitative data from both patients and professionals experiencing the intervention.

### Health economics analysis

The objective of the economic study will be to assess the relative cost-effectiveness of the training intervention to improve patient outcomes and/or the costs of health and social care. The framework of cost-effectiveness and the cost-effectiveness acceptability analysis will be used. The perspective of the study will include the viewpoints of health and social care service providers and patients. These are thought to be the key actors and thus approximate a societal perspective. Costs and outcomes will be discounted at the rate recommended by the UK Treasury at the time of analysis (currently 3.5%).

#### Outcome measures

The primary outcomes of the economic evaluation will be the incremental cost-effectiveness ratio (ICER) and the associated net benefit statistic and probability of cost-effectiveness derived from the cost-effectiveness acceptability analysis. The measure of patient outcome for the economic analysis will be quality-adjusted life years (QALYs) gained at the end of scheduled follow-up. The QALYs will be estimated as the length of time from baseline to end of follow-up, multiplied by the utility of that period. The utility values of the participants will be estimated from the EQ-5D health status questionnaire [66] completed at baseline and follow-up assessments, and the associated published societal utility tariffs.

#### Costs

The direct costs will include the costs of the training intervention and the costs of health and social care services used from recruitment to the end of the scheduled follow-up. The direct costs of treatment and subsequent care will be estimated by summing the costs of each resource used as an input to provide health and social care. For each type of resource, the cost will be estimated as the quantity of that resource used multiplied by the unit cost specific to that resource. In addition, previous health and social care costs are known determinants of future costs. Information will be collected from participants and primary-care records about service use for the 6 months prior to baseline, and these costs will be used as a covariate in the economic data analysis.

The analysis of economic data will use an intent-to-treat approach, and missing data will be imputed. Missing observations will be assumed to be missing at random and imputed using multiple imputations. Censored data due to death or withdrawal for other reasons will be imputed using survival analysis.

ICERs will be estimated as cost per QALY gained for the primary and sensitivity analyses. Analysis of covariance (ANCOVA) will be used to estimate the net costs and QALYs associated with the training intervention to adjust for any differences in baseline characteristics of patients or practices. The cost-effectiveness acceptability analysis will be generated from the ANCOVA to estimate the probability that an intervention is cost-effective [93]. The cost-effectiveness acceptability analysis will be used to estimate the probability that an intervention is cost-effective using ceiling thresholds in the range £0 per QALY gained to £30000 per QALY gained, in increments of £1000. Bootstrap estimates of the ICER and net benefit statistic (mean 2.5th to 97.5th percentile), cost-effectiveness plane and cost acceptability curve will be presented [89,90].

Sensitivity analysis will be used to test the effects of assumptions and data on the ICER, and results of the cost-effectiveness acceptability analysis. These will include testing the effects of varying the discount rate used, extrapolating the results over longer timeframes, the unit cost sets used to value resources, the methods used to impute missing data; and the outcome measure used in the ICER including 1) severity of depressive symptoms (SCL-90, PHQ-9), 2) health perceptions (IPQ), 3) self-management behaviors, and 4) other measures of health status.

SPSS Statistics (IBM, version 20.0) will be used for data manipulation and descriptive analysis. Stata 12 (StataCorp, Texas 77845–4512, USA) will be used for the main statistical analyses, and imputation and estimation of net benefit statistics and cost-effectiveness acceptability analysis [93,94].

### Analysis

The method of allocation of practices to the collaborative care or control arm will be based on simple randomization (that is, 50:50 chance) for the first six practices. Minimization will be used thereafter with 0.7/0.3 probabilities. For each allocation, the balance score will be estimated based on thresholds for practice list size and deprivation, measured using the Index of Multiple Deprivation.

All analyses will be conducted on an intention-to-treat basis subject to the availability of data. After data cleaning, the preliminary analysis will investigate the pattern of missing baseline and follow-up data. The effect of the collaborative-care treatment program on depression outcomes will be estimated using a multi-level mixed-effects linear regression model, including a random effect for practices, and controlling for the following baseline covariates: patient age, gender, ethnicity, and baseline PHQ-9 score. Given that the continuous outcomes are likely to have a skewed distribution, bootstrap sampling (a method free from parametric assumptions) will be used to derive estimates of error variance for the tests of statistical significance, using 10,000 samples. Sensitivity analysis will be carried out to check the implications of different assumptions about the missing data.

### Ethics

The study has received ethics approval from the National Research Ethical Service (NRES) NRES Committee North West Preston (REC code 11/NW/0742). It will be conducted in accordance with the UK Department of Health Research Governance Framework in health and social, care and adhere to the ethical principles of the Helsinki Declaration [95]. All research staff involved in the conduct of the trial will meet the standards laid out in the *ICH Harmonized Tripartite Guideline for Good Clinical Practice*[96].

No patient participant will be offered financial incentives to take part. Professionals invited to take part in qualitative interviews may be reimbursed for their time. In addition, all data will be anonymized and secured off site for a period of at least 5 years in accordance with the Data Protection Act 1995.

The design of this trial will not disadvantage patients in either the collaborative-care or control groups. No treatment is being withheld from patients in the control group, and patients in this group will still be eligible to receive evidence-based treatment for depression. After completion of follow-ups and the ‘train the trainer sessions’, control practices will have access to low-intensity therapists trained in collaborative care for depression and LTCs. The design therefore approximates a wait-list control design, which is ethically more acceptable than a no-treatment control design.

### User engagement

Service users will be recruited from voluntary self-help groups for people with diabetes and/or CHD across Greater Manchester, and will be involved with key aspects of the trial, including: 1) the trial steering committee (TSC); 2) the data analysis stage of the qualitative process evaluation; and 3) in ensuring information disseminated to patients and healthcare professionals during participant recruitment is relevant, appropriate and informative. Service users will be provided with training and support where necessary, and travel costs and time commitments for attending team meetings will be reimbursed at a rate commensurate with good clinical practice in the NHS [97]. Representatives will be advised that their participation is on a voluntary basis, and will be assured that any information about the trial fed back to the team will be confidential.

### The trial steering committee and the data monitoring and ethics committee

Independent supervision of the trial will be carried out by members of the TSC. The TSC will have responsibility for monitoring progress of the trial, adherence to the protocol, patient safety, and consideration of new information. Membership of the TSC will include the principal investigator along with an independent chair, and at least two other independent members. The trial manager and the trial statistician will attend when appropriate. An observer from the host institution (University of Manchester) will be invited to attend the TSC.

The data monitoring committee and ethics committee (DMEC) will be the only body in the trial with full access to unblinded comparative data. Members of the DMEC will be completely independent from the trial and the host institution, and will report to the TSC to determine if there are ethical or safety reasons for the trial not to continue.

### Forecast execution dates

The set-up period began in August 2011, and continued until December 2011. Recruitment of GP practices and IAPT teams began in January 2012 and continued in two sequential waves for 6 months. Patient recruitment started in May 2012 and will continue until January 2013. Follow-up will start in November 2012 and continue until all patients have been assessed at 6 months. Data analysis will start in August 2013.

## Discussion

Developing new systems to deliver care for depression and for depression plus LTCs remains a significant challenge for the future. It is still unclear how integrated care models can be disseminated and implemented in routine care in settings in which limited resources, professional resistance, and competing priorities are significant barriers to change, barriers likely to be exacerbated in these times of economic uncertainty. The COINCIDE trial aims to train PWPs to deliver the benefits of integrated care to patients with co-morbid depression and diabetes/CHD. These professionals have the advantage of an existing knowledge base around the delivery of evidence-based brief psychological treatments. Our challenge is to provide them with skills to deliver these treatments, in partnership with primary-care staff, to patients with LTCs, and to overcome some of the complex interactions between mental and physical health that can stand in the way of patient change [98]. In addition, by harnessing and enhancing the skills of usual care providers employed by IAPT services the COINCIDE trial offers a unique opportunity to establish durable organizational change in the way in which patients with depression and LTCs are managed in primary care.

Populations with depression and co-occurring multiple medical problems rarely get access to collaborative care, even though the evidence that collaborative care may be effective in adults with depression and LTCs is accumulating [25]. We anticipate that the results of this trial will further support the case for delivering collaborative models of care to patients with common mental-health problems and LTCs, thus improving access and quality of mental-health services for under-served groups.

## Abbreviations

ANCOVA: Analysis of covariance; BA: Behavioral activation; BME: Black and minority and ethic; CBT: Cognitive behavioral therapy; CHD: Coronary heart disease; CLAHRC: Collaboration for Leadership in Applied Health Research and Care; COINCIDE: Collaborative Interventions for Circulation and Depression; CSO: Clinical studies officer; CSQ-8: Client Satisfaction Questionnaire-8; DMEC: Data monitoring and ethics committee; DNA: Did not attend; DQoL: Diabetes Quality of Life; ENRICHD: Enhancing Recovery in Coronary Heart Disease; EQ-5D: EuroQoL 5-D; GAD-7: Generalized Anxiety Disorder Assessment-7; GM: Greater Manchester; GP: General Practitioner; IAPT: Increasing Access to Psychological Therapies; ICER: Incremental cost-effectiveness ratio; LTC: Long-term condition; MHRN: Mental Health Research Network; NHS: National Health Service; NICE: National Insitute for Health and Clinical Excellence; NPT: Normalization process theory; NRES: National Research Ethics Service; PACIC-5A: Patient Assessment of Chronic Illness Care; PHQ-9: Patient Health Questionnaire-9; PWP: Psychological well-being practitioner; QALY: Quality-adjusted life year; QOF: Quality and Outcomes Framework; RCT: Randomized controlled trial; RSQ: Relationship Scales Questionnaire; SAQ: Seattle Angina Questionnaire; SCL-90: Symptoms Checklist-90; TSC: Trial steering committee; WHOQoL-BREF: World Health Organization Quality of Life brief measure.

## Competing interests

PC, CG, CJG, CK, IA, KR, AC, FJ, CD, CCG declare they have no competing interests. PB is a paid scientific consultant to the British Association of Counselling and Psychotherapy.

## Authors contributions

PC is the chief investigator, designed the study and wrote the first draft of the protocol, and revised and edited subsequent versions for publication. LG is the co-lead of the program, contributed to study design, and co-authored the manuscript. CD contributed to study design and outcome selection and edited the manuscript. PB contributed to study design and co-authored the manuscript. CB contributed to the design of the training program and edited the manuscript. C CG contributed to the design of the process evaluation and edited the manuscript. CG contributed to the design of the training program and edited the manuscript. CJG prepared the manuscript for publication and assisted with trial design and selection of outcome measures. KL co-authored the manuscript and contributed to the design of the training program. IA contributed to the design of the recruitment strategy and edited the manuscript. KR contributed to writing sections on therapeutic interventions and edited the manuscript. CK contributed to the writing the user engagement section and edited the manuscript. WW contributed to the design of the recruitment strategy for South Asian patients and the translation of outcome measures. MH performed the sample size calculation and edited the manuscript. LD contributed to writing the health economic evaluation and edited the manuscript. FJ contributed to writing the health economic evaluation and edited the manuscript. CR contributed to the statistical analysis of the study. SK contributed to writing the process evaluation section. AC contributed to study design and is the trial manager. All authors edited the manuscript and read and approved the final manuscript.
